# A Comprehensive Review of Metabolic Syndrome and Its Role in Cardiovascular Disease and Type 2 Diabetes Mellitus: Mechanisms, Risk Factors, and Management

**DOI:** 10.7759/cureus.67428

**Published:** 2024-08-21

**Authors:** Rushikesh H Dhondge, Sachin Agrawal, Rajvardhan Patil, Ajinkya Kadu, Manjeet Kothari

**Affiliations:** 1 General Medicine, Jawaharlal Nehru Medical College, Datta Meghe Institute of Higher Education and Research, Wardha, IND

**Keywords:** dyslipidemia, obesity, insulin resistance, type 2 diabetes mellitus, cardiovascular disease, metabolic syndrome

## Abstract

Metabolic syndrome is a multifaceted metabolic disorder characterized by a constellation of interconnected risk factors, including insulin resistance, abdominal obesity, dyslipidemia, and hypertension. These components collectively predispose individuals to an elevated risk of cardiovascular disease (CVD) and type 2 diabetes mellitus (T2DM). The prevalence of metabolic syndrome has escalated globally, paralleling the rise in obesity rates and sedentary lifestyles. This review explores the pathophysiology underlying metabolic syndrome, emphasizing its role in the development and progression of CVD and T2DM. Epidemiological data underscore the substantial public health burden metabolic syndrome poses, necessitating effective preventive strategies and management approaches. The current diagnostic criteria and screening tools are discussed, highlighting their utility in clinical practice. Management strategies encompass lifestyle modifications, pharmacotherapy, and surgical interventions, each targeting specific components of metabolic syndrome to mitigate cardiovascular and metabolic risks. The challenges in diagnosing and managing metabolic syndrome are addressed alongside emerging research directions to enhance prevention and treatment outcomes. By elucidating the intricate relationship between metabolic syndrome, CVD, and T2DM, this review aims to guide healthcare practitioners in optimizing patient care and advancing public health initiatives to combat this pervasive syndrome.

## Introduction and background

Metabolic syndrome represents a cluster of interconnected metabolic abnormalities that significantly increase the risk of cardiovascular disease (CVD) and type 2 diabetes mellitus (T2DM) [1]. This syndrome is characterized by insulin resistance or glucose intolerance, abdominal obesity, dyslipidemia (elevated triglycerides, low high-density lipoprotein (HDL) cholesterol levels), and hypertension [2]. The presence of any three out of these five components typically qualifies an individual as having metabolic syndrome. Various organizations, including the National Cholesterol Education Program (NCEP) and the International Diabetes Federation (IDF), have established criteria for diagnosing metabolic syndrome, which may vary slightly but generally emphasizes the same core risk factors [3]. Metabolic syndrome has reached epidemic proportions globally, posing a significant public health challenge. Its prevalence varies across populations and is influenced by age, ethnicity, and lifestyle choices [4]. In the United States, approximately one-third of adults are estimated to have metabolic syndrome, with prevalence rates increasing with age. Similar trends are observed worldwide, particularly in urbanized and developed regions where sedentary lifestyles and high-calorie diets are more common. The increasing prevalence parallels rising rates of obesity and sedentary lifestyles, highlighting the urgent need for effective public health interventions [5].

Understanding metabolic syndrome is crucial due to its strong association with an elevated risk of developing CVD, including coronary artery disease, stroke, and peripheral vascular disease, as well as T2DM. Individuals with metabolic syndrome are at higher risk of experiencing adverse cardiovascular events and complications associated with diabetes [6]. The syndrome's components, such as insulin resistance and dyslipidemia, directly contribute to the pathogenesis of atherosclerosis and chronic hyperglycemia, which are fundamental processes in CVD and T2DM, respectively. Consequently, addressing metabolic syndrome can significantly reduce the burden of these chronic diseases [7]. This review aims to comprehensively examine the mechanisms, risk factors, and management strategies associated with metabolic syndrome. By synthesizing current literature and clinical evidence, this review aims to provide a holistic understanding of the complex interplay between metabolic syndrome, CVD, and T2DM, highlighting implications for clinical practice and public health initiatives. This review will also identify gaps in existing research and suggest future directions for investigation, aiming to inform better prevention and treatment strategies for metabolic syndrome and its associated conditions.

## Review

Pathophysiology and mechanisms

The pathophysiology of metabolic syndrome is intricate, involving multiple interrelated mechanisms contributing to its development and progression. One of the primary factors is insulin resistance, which impairs glucose uptake by cells and increases hepatic glucose production [1]. This results in hyperglycemia, prompting the pancreas to compensate by producing more insulin, leading to a condition known as hyperinsulinemia. Although this compensatory mechanism initially helps maintain normal glucose levels, it can eventually adversely affect lipid metabolism and blood pressure regulation [8]. Central obesity plays a crucial role in the development of metabolic syndrome. Excessive visceral fat accumulation is particularly harmful, leading to adipose tissue dysfunction. This dysfunction is characterized by an increased release of free fatty acids and pro-inflammatory cytokines alongside a decrease in the secretion of protective factors like adiponectin. Dysfunctional adipose tissue contributes to insulin resistance, atherogenic dyslipidemia, and hypertension, creating a vicious cycle that exacerbates the syndrome [9]. Dyslipidemia is another key feature of metabolic syndrome, marked by elevated triglycerides and low levels of HDL cholesterol. Insulin resistance impairs the ability of insulin to suppress hepatic production of very low-density lipoprotein (VLDL), leading to increased triglyceride levels. Concurrently, low HDL cholesterol levels are often observed, likely due to increased activity of cholesteryl ester transfer protein (CETP) and reduced maturation of HDL particles. This dyslipidemic profile significantly heightens the risk of CVD [10]. Hypertension is also a common component of metabolic syndrome, largely driven by the interplay of insulin resistance and hyperinsulinemia. These conditions stimulate the sympathetic nervous system and activate the renin-angiotensin-aldosterone system, contributing to elevated blood pressure. Additionally, insulin resistance can lead to increased sodium reabsorption and impaired vasodilation, further exacerbating hypertension [11]. Pro-inflammatory and pro-thrombotic states are another critical aspect of metabolic syndrome. Excess visceral fat secretes pro-inflammatory cytokines, such as tumor necrosis factor-alpha (TNF-α) and interleukin-6 (IL-6), contributing to a chronic inflammatory environment. This inflammation, coupled with insulin resistance, promotes a pro-thrombotic state by increasing plasminogen activator inhibitor-1 (PAI-1) and fibrinogen levels, heightening the risk of clot formation and cardiovascular events [12]. Finally, genetic and environmental factors play significant roles in the development of metabolic syndrome. Genetic predisposition can influence an individual's susceptibility to the syndrome and its components. Meanwhile, environmental factors such as a sedentary lifestyle, unhealthy dietary habits, and chronic stress can interact with genetic vulnerabilities, further increasing the risk of metabolic syndrome. Understanding the complex interplay between these mechanisms is crucial for developing effective prevention and treatment strategies for metabolic syndrome and its associated conditions, such as CVD and T2DM [13].

Risk factors

**Figure 1:**
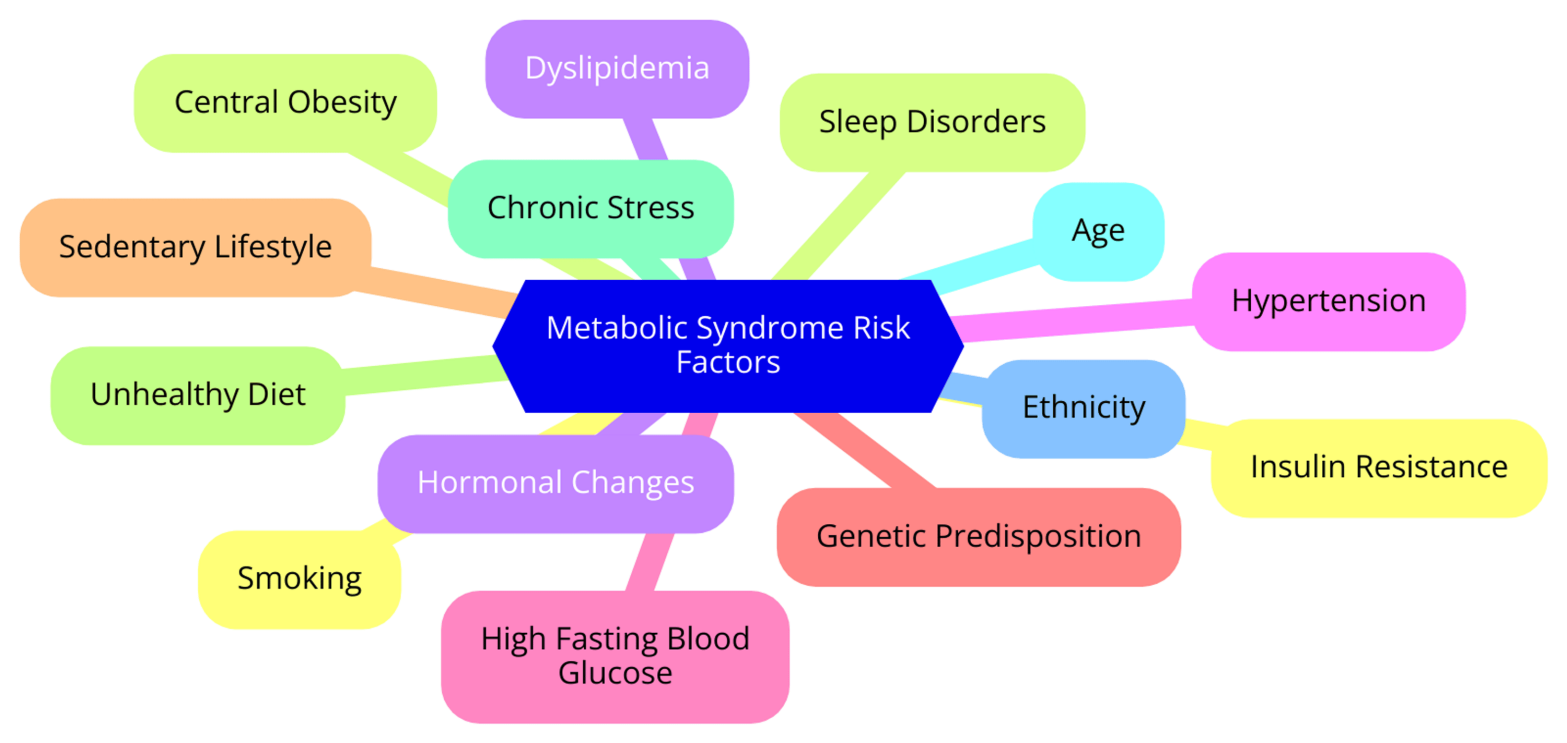
Risk factors for metabolic syndrome Image credit: Dr Rushikesh H. Dhondge

Metabolic syndrome is a complex condition characterized by a cluster of interrelated risk factors that significantly increase the risk of developing CVD and T2DM [14]. The key components of metabolic syndrome include abdominal obesity, high blood pressure, impaired fasting glucose, high triglycerides, and low HDL cholesterol. It is closely linked to insulin resistance, which hinders cells from responding effectively to insulin and leads to elevated blood glucose levels. Obesity, a sedentary lifestyle, genetic factors, and chronic stress are major contributors to the development of metabolic syndrome [14]. Individuals with metabolic syndrome are at a much higher risk of developing T2DM compared to those without it. The combination of insulin resistance, atherogenic dyslipidemia, hypertension, a prothrombotic state, and endothelial dysfunction in metabolic syndrome significantly raises the risk of CVD, even in the absence of glucose intolerance. The prevalence of metabolic syndrome has reached alarming levels worldwide, with certain ethnic groups, such as African Americans and Mexican Americans, being at higher risk [15]. The risk factors for metabolic syndrome can be divided into non-modifiable and modifiable categories. Non-modifiable risk factors include age, gender, and genetic predisposition. The risk of metabolic syndrome increases with age, and women generally have a higher risk, especially after menopause. Certain genetic factors may also increase an individual's susceptibility to developing metabolic syndrome and its components [16]. Modifiable risk factors, on the other hand, can be addressed through lifestyle changes and interventions. Diet and nutrition play a crucial role, with a diet high in processed foods, refined carbohydrates, and unhealthy fats contributing to the development of metabolic syndrome. Regular physical activity helps maintain a healthy weight, improves insulin sensitivity, and reduces the risk of metabolic syndrome [17]. Smoking is a significant risk factor for metabolic syndrome and is associated with an increased risk of CVD and T2DM. Excessive alcohol intake can lead to weight gain, hypertension, and dyslipidemia, which are components of metabolic syndrome. Chronic sleep deprivation and high levels of stress can disrupt hormonal balance, increase inflammation, and contribute to the development of metabolic syndrome [18]. Early identification and management of metabolic syndrome are essential to prevent or delay the onset of T2DM and CVD. The primary and first-choice therapies are lifestyle modifications, including weight loss, increased physical activity, and dietary changes. Pharmacological treatment may be necessary for managing individual components of metabolic syndrome, such as hypertension, dyslipidemia, and hyperglycemia. By addressing these risk factors through a comprehensive approach, individuals can significantly reduce their risk of developing metabolic syndrome and its associated complications [19]. The risk factors are shown in Figure 1.

Metabolic syndrome and cardiovascular disease

Metabolic syndrome is a significant risk factor for CVD, with various mechanisms linking the two conditions. It contributes to atherosclerosis through several pathways, including insulin resistance, inflammation, and oxidative stress. Insulin resistance leads to dyslipidemia, elevated triglycerides, and low HDL cholesterol levels, promoting atherogenic processes [20]. Additionally, chronic low-grade inflammation associated with metabolic syndrome results in endothelial dysfunction, impaired vasodilation, and increased arterial stiffness, further exacerbating atherosclerosis. Elevated levels of pro-inflammatory cytokines such as IL-6 and TNF-α are common in individuals with metabolic syndrome. These cytokines contribute to vascular inflammation and damage, promoting the progression of atherosclerosis and increasing the risk of CVD events. Excessive reactive oxygen species (ROS) in metabolic syndrome leads to oxidative stress, which damages endothelial cells and promotes plaque formation in arteries. This oxidative environment can also modify lipoproteins, such as oxidized low-density lipoproteins (LDL), which are particularly atherogenic [21]. Individuals with metabolic syndrome have a significantly increased risk of myocardial infarction (MI) and stroke. Studies indicate that the presence of metabolic syndrome doubles the risk of CVD events, including MI and stroke, compared to those without the syndrome [22]. The mechanisms include increased thrombotic risk, as metabolic syndrome is associated with a hypercoagulable state, increasing the likelihood of thrombus formation that can lead to MI or stroke. Epidemiological studies have shown that patients with metabolic syndrome experience higher rates of acute coronary syndromes and cerebrovascular accidents, largely due to the underlying atherosclerotic changes and endothelial dysfunction [23]. The link between metabolic syndrome and heart failure is also well-established. The syndrome's components, such as hypertension and diabetes, contribute to left ventricular hypertrophy and diastolic dysfunction, ultimately leading to heart failure. The risk of developing heart failure is particularly pronounced in individuals with both metabolic syndrome and pre-existing CVD [24]. Numerous studies have demonstrated the strong association between metabolic syndrome and CVD. Research indicates that metabolic syndrome is associated with a two-fold increase in the risk of CVD and a 1.5-fold increase in all-cause mortality. The prevalence of metabolic syndrome is rising globally, paralleling the increase in obesity and sedentary lifestyles, which further compounds the risk of CVD. Long-term studies, such as the Framingham Heart Study, have consistently shown that metabolic syndrome is a strong predictor of future cardiovascular events, even in individuals without diabetes [25].

Metabolic syndrome and type 2 diabetes mellitus

Metabolic syndrome significantly increases the risk of developing T2DM through several key mechanisms. Insulin resistance, a hallmark of metabolic syndrome, leads to impaired glucose tolerance and increased insulin secretion by pancreatic beta cells. Over time, chronic hyperinsulinemia and insulin resistance can cause beta cell failure, possibly due to glucose and lipid toxicity, ultimately resulting in T2DM [26]. Obesity, a major component of metabolic syndrome, is associated with chronic inflammation and increased production of pro-inflammatory factors like TNF-α. This inflammatory state contributes to insulin resistance and beta-cell dysfunction. Oxidative stress, an imbalance between free radical production and antioxidant defenses, is also linked to the pathogenesis of insulin resistance in metabolic syndrome. Excess reactive oxygen species can impair insulin signaling and glucose homeostasis [27]. Increased visceral and ectopic fat accumulation, particularly in the liver and skeletal muscle, is a key feature of metabolic syndrome. Elevated levels of non-esterified fatty acids and triglycerides can lead to lipotoxicity, further exacerbating insulin resistance and beta cell dysfunction. Epidemiological studies consistently show that individuals with metabolic syndrome are five times more likely to develop T2DM compared to those without it. The prevalence of metabolic syndrome has reached alarming levels worldwide, with almost half a billion people living with diabetes globally [28,29].

Diagnosis and screening

Diagnosis and screening for metabolic syndrome are crucial for identifying individuals at increased risk for CVD and T2DM. Several established guidelines help clinicians diagnose metabolic syndrome, with the National Cholesterol Education Program Adult Treatment Panel III (NCEP ATP III) criteria being widely recognized. According to these guidelines, metabolic syndrome is diagnosed when a patient meets at least three of the following five conditions: abdominal obesity (waist circumference ≥102 cm in men and ≥88 cm in women), elevated triglycerides (≥150 mg/dL or treatment for high triglycerides), reduced HDL cholesterol (<40 mg/dL in men and <50 mg/dL in women or treatment for low HDL), high blood pressure (≥130/85 mm Hg or treatment for hypertension), and elevated fasting glucose (≥100 mg/dL or treatment for high blood sugar) [7]. The IDF has its diagnostic criteria that prioritize abdominal obesity as a prerequisite for diagnosis, allowing for alternative definitions based on body mass index (BMI) in specific populations [30]. The World Health Organization (WHO) focuses on insulin resistance as a key component, requiring evidence of insulin resistance and other metabolic abnormalities to diagnose metabolic syndrome [31]. This approach underscores the syndrome's complexity and deep-seated relationship with metabolic health. Screening for metabolic syndrome typically involves a combination of physical examinations and laboratory tests. Essential methods include measuring waist circumference to assess abdominal obesity, conducting blood tests to evaluate levels of triglycerides, HDL cholesterol, and fasting glucose, and measuring blood pressure. A comprehensive assessment of medical history and potential symptoms related to metabolic syndrome, such as fatigue or signs of insulin resistance, is also crucial for accurate diagnosis [32]. Early detection of metabolic syndrome is critical due to its strong association with increased risks of CVD and T2DM. Identifying individuals with metabolic syndrome allows for prompt interventions, including lifestyle modifications such as dietary changes, increased physical activity, and appropriate medical treatments [33]. Research indicates that individuals with metabolic syndrome face double the risk of developing coronary artery disease and an elevated likelihood of progressing to diabetes and other chronic conditions. Therefore, early diagnosis is essential for implementing preventive measures and improving long-term health outcomes, ultimately reducing the burden of these serious health issues [34].

Management and treatment strategies

Effective management of metabolic syndrome is essential for reducing the risk of CVD and T2DM. This comprehensive approach includes lifestyle modifications, pharmacological treatments, surgical interventions, and consideration of emerging therapies [35]. Lifestyle changes are foundational in managing metabolic syndrome. Dietary interventions are crucial, focusing on a balanced diet rich in fruits, vegetables, whole grains, lean proteins, and healthy fats. Diets such as the Mediterranean and DASH (Dietary Approaches to Stop Hypertension) diets are often recommended. Monitoring caloric intake is important for promoting weight loss and maintaining a healthy weight while limiting processed foods, sugar-sweetened beverages, and trans fats. Portion control and mindful eating practices can further support weight management and prevent overeating [36]. Physical activity guidelines suggest engaging in at least 150 minutes of moderate-intensity aerobic exercise per week, such as brisk walking or cycling, along with muscle-strengthening activities two or more days per week. Increasing daily activity - such as walking more or taking the stairs - also contributes to overall health. Weight loss is critical, with a target of a 5-10% reduction in body weight, significantly improving metabolic parameters. Behavioral strategies, including goal setting, self-monitoring, and support groups, can aid in achieving and maintaining weight loss [37]. Pharmacological treatments may be necessary for managing specific components of metabolic syndrome. First-line insulin resistance and glycemic control treatments include antihyperglycemic agents such as metformin. Glucagon-like peptide 1 (GLP-1) receptor agonists and sodium-glucose transport protein 2 (SGLT2) inhibitors also effectively lower blood glucose levels while providing cardiovascular benefits. Statins are commonly prescribed for dyslipidemia to reduce LDL cholesterol and lower cardiovascular risk, while fibrates can help decrease triglyceride levels and increase HDL cholesterol. Antihypertensive medications, including angiotensin-converting-enzyme (ACE) inhibitors, angiotensin receptor blockers (ARBs), and calcium channel blockers, effectively manage hypertension and offer additional benefits for metabolic health [38]. In certain cases, surgical interventions may be appropriate, especially bariatric surgery. This option is typically recommended for individuals with a BMI of 35 or higher who have not achieved significant weight loss through lifestyle changes. Procedures such as gastric bypass, sleeve gastrectomy, and adjustable gastric banding can lead to substantial weight loss, improved metabolic parameters, and a reduced incidence of T2DM and CVD [39]. Emerging therapies and future directions in the management of metabolic syndrome are promising. Research into novel pharmacotherapies targeting insulin resistance, inflammation, and lipid metabolism is ongoing. Personalized medicine approaches may tailor interventions based on genetic and phenotypic profiles, enhancing treatment efficacy. Additionally, the rise of digital health solutions, including apps and wearable technology, enables better monitoring of physical activity, diet, and metabolic parameters. Enhanced behavioral interventions, particularly those employing cognitive-behavioral strategies, may also improve adherence to lifestyle modifications [40].

Prevention strategies

Preventing metabolic syndrome is vital for reducing the incidence of T2DM and CVD. A comprehensive strategy involving public health interventions, community-based programs, and policy changes can significantly enhance prevention efforts. Public health interventions should focus on health education campaigns to raise awareness about metabolic syndrome's risk factors and consequences, utilizing various media platforms to reach diverse populations. Screening initiatives in healthcare settings can help identify at-risk individuals early [41]. Nutritional guidelines should advocate for a balanced diet rich in fruits, vegetables, whole grains, and lean proteins while minimizing the intake of processed foods, sugars, and unhealthy fats. Clear food labeling can assist consumers in making informed dietary choices. Promoting physical activity is also crucial; programs encouraging regular exercise, such as walking groups, fitness classes, and workplace wellness initiatives, are essential. Infrastructure improvements to support active transportation, such as walking and cycling, should be prioritized [42]. Community-based programs are instrumental in prevention efforts. Local health initiatives, including community health fairs that offer free screenings, nutritional advice, and physical activity demonstrations, can directly engage the community. Support groups for individuals at risk of metabolic syndrome provide platforms for sharing experiences and strategies for lifestyle changes. School-based programs should incorporate nutrition education into curricula to teach children about healthy eating habits early on and ensure regular physical activity opportunities such as recess, sports, and after-school programs. Workplace wellness programs that offer incentives for employees to engage in healthy behaviors, such as gym memberships or health screenings, can foster a culture of wellness. Additionally, stress management workshops can provide resources for managing chronic stress, which contributes to metabolic syndrome [43]. Policy changes and health promotion are essential for creating environments conducive to prevention. Legislative actions should focus on food policy reforms to limit the availability of unhealthy options in schools and public institutions while promoting access to healthy foods. Stricter regulations on tobacco use, given its role as a risk factor for metabolic syndrome, should be enforced. Urban planning should prioritize the creation of green spaces and parks to encourage physical activity, and improvements in public transportation can reduce reliance on cars and promote walking and cycling. Ensuring affordable healthcare services, including preventive care and management of chronic conditions, is crucial for healthcare access and equity. Culturally relevant programs tailored to diverse populations' needs can help address health disparities [44]. Effective prevention of metabolic syndrome requires a multifaceted approach that combines public health initiatives, community engagement, and supportive policy changes. By creating environments that foster healthy lifestyles and addressing the social determinants of health, we can significantly reduce the prevalence of metabolic syndrome, thereby lowering the risk of T2DM and CVD. Collaboration among healthcare providers, community organizations, policymakers, and individuals is essential to achieving these objectives [45].

Challenges and controversies

The challenges and controversies surrounding metabolic syndrome involve various dimensions, including diagnostic criteria, debates about its existence, healthcare disparities, and long-term management [46]. A primary challenge is the significant variability in diagnostic criteria for metabolic syndrome. Different organizations, such as the National Cholesterol Education Program (NCEP), IDF, and WHO, have proposed distinct definitions for metabolic syndrome. This lack of standardization leads to discrepancies in prevalence rates and risk assessments across populations. Comparative analyses have shown that, while most definitions correlate with increased cardiovascular risk, the specific criteria used can result in varying diagnostic outcomes, complicating identifying and managing individuals at risk [47]. The existence and definition of metabolic syndrome have also been subjects of ongoing debate among healthcare professionals. Critics argue that the syndrome is too broad and may lead to overdiagnosis, suggesting it could pathologize normal variations in metabolic health. Conversely, proponents emphasize the utility of metabolic syndrome in identifying individuals at high risk for CVD and T2DM. Some researchers question the need for a distinct syndrome altogether, proposing that focusing on individual risk factors might be more relevant for clinical management than categorizing patients under a broad umbrella [4]. Healthcare disparities further complicate the management of metabolic syndrome. Individuals from lower socioeconomic backgrounds often face significant barriers to accessing healthcare services, leading to underdiagnosis and inadequate management of the syndrome. This issue is particularly concerning in marginalized communities, where higher rates of complications, such as CVD and diabetes, are observed. Addressing these disparities is crucial for improving outcomes and ensuring equitable healthcare for all populations [48]. Finally, long-term management of metabolic syndrome presents significant challenges, particularly regarding adherence to lifestyle modifications and pharmacological treatments. Many patients struggle to maintain dietary changes, engage in regular physical activity, and adhere to medication regimens, all of which are essential for managing metabolic syndrome components. Lack of support, education, and resources can hinder adherence, ultimately affecting health outcomes and increasing the risk of associated diseases [49].

Future research directions

As our understanding of metabolic syndrome advances, several key areas require further exploration to improve prevention, diagnosis, and management strategies. One promising avenue is the identification of novel biomarkers. Discovering new biomarkers can enhance early detection and monitoring of metabolic syndrome and its associated complications. Researchers can leverage advanced technologies such as proteomics, metabolomics, and genomics to identify specific proteins, metabolites, or genetic markers linked to metabolic syndrome. Additionally, studying inflammatory markers, adipokines, and gut microbiota profiles may reveal indicators of metabolic dysfunction. Early identification of at-risk individuals can enable timely interventions, potentially reversing or halting the progression of metabolic syndrome [40]. Another critical area for future research involves understanding the genetic and epigenetic influences on metabolic syndrome. Investigating how genetic predispositions and epigenetic modifications contribute to the development of metabolic syndrome can provide valuable insights. Genome-wide association studies (GWAS) can help identify genetic variants associated with metabolic syndrome. Furthermore, exploring the role of epigenetic factors, such as DNA methylation and histone modification, in metabolic regulation and responses to environmental factors like diet and stress can deepen our understanding of the condition. This knowledge could lead to personalized prevention and treatment strategies, tailoring interventions based on an individual’s genetic and epigenetic profile [50]. Exploring new therapeutic targets represents another promising research direction. Identifying and validating new therapeutic targets for treating metabolic syndrome and its complications could significantly improve clinical outcomes. Researchers should investigate novel pathways involved in insulin signaling, inflammation, and lipid metabolism. Additionally, examining the potential of pharmacological agents that target specific metabolic pathways, such as GLP-1 receptor agonists, SGLT2 inhibitors, and innovative anti-inflammatory drugs, may lead to the development of more effective treatments. These new therapies could enhance the effectiveness of current regimens and provide alternatives for patients who do not respond to existing medications [51]. Finally, conducting longitudinal studies on prevention and management outcomes is essential for understanding the long-term effectiveness of various strategies for metabolic syndrome. Large-scale cohort studies tracking lifestyle interventions, pharmacological treatments, and their impacts on metabolic syndrome progression and related health outcomes will be invaluable. Researchers should assess the long-term effects of lifestyle changes, such as diet and exercise, on cardiovascular health and the incidence of type 2 diabetes. Longitudinal data can provide insights into the sustainability of interventions, inform public health strategies, and guide clinical practice in managing metabolic syndrome [52].

## Conclusions

In conclusion, metabolic syndrome is a significant and growing public health concern, intimately linked with an increased risk of CVD and T2DM. This cluster of metabolic abnormalities - including insulin resistance, central obesity, dyslipidemia, and hypertension - constitutes a major contributor to morbidity and mortality worldwide. Understanding the mechanisms and risk factors underlying metabolic syndrome is crucial for developing effective prevention and management strategies. Lifestyle modifications, pharmacological interventions, and public health initiatives play critical roles in mitigating the impact of metabolic syndrome on individual and population health. Comprehensive management approaches that address the multifaceted nature of metabolic syndrome can reduce the incidence of CVD and T2DM, ultimately improving health outcomes and quality of life for affected individuals. Continued research is essential to refine our understanding of metabolic syndrome and to develop innovative therapies and interventions. We can make significant strides in combating this pervasive syndrome and its associated complications through concerted efforts in research, clinical practice, and public health policy.

## References

[1] Rochlani Y, Pothineni NV, Kovelamudi S, Mehta JL (2017). Metabolic syndrome: pathophysiology, management, and modulation by natural compounds. Ther Adv Cardiovasc Dis.

[2] (2024). Metabolic syndrome. https://www.hopkinsmedicine.org/health/conditions-and-diseases/metabolic-syndrome.

[3] Shrivastava S, Singh K, Chakma T, Kavishwar A (2023). Metabolic syndrome in Indian tribes: challenges to reveal its true status. Front Clin Diabetes Healthc.

[4] Swarup S, Ahmed I, Grigorova Y, Zeltser R (2024). Metabolic syndrome. StatPearls.

[5] Saklayen MG (2018). The global epidemic of the metabolic syndrome. Curr Hypertens Rep.

[6] Whayne TF (2010). Metabolic syndrome, peripheral vascular disease and coronary artery disease: a concise review. Int J Angiol.

[7] Ginsberg HN, MacCallum PR (2009). The obesity, metabolic syndrome, and type 2 diabetes mellitus pandemic: Part I. Increased cardiovascular disease risk and the importance of atherogenic dyslipidemia in persons with the metabolic syndrome and type 2 diabetes mellitus. J Cardiometab Syndr.

[8] Petersen MC, Shulman GI (2018). Mechanisms of insulin action and insulin resistance. Physiol Rev.

[9] Kawai T, Autieri MV, Scalia R (2021). Adipose tissue inflammation and metabolic dysfunction in obesity. Am J Physiol Cell Physiol.

[10] Hirano T (2018). Pathophysiology of diabetic dyslipidemia. J Atheroscler Thromb.

[11] Zhou MS, Wang A, Yu H (2014). Link between insulin resistance and hypertension: what is the evidence from evolutionary biology?. Diabetol Metab Syndr.

[12] Sypniewska G (2007). Pro-inflammatory and prothrombotic factors and metabolic syndrome. EJIFCC.

[13] Abou Ziki MD, Mani A (2016). Metabolic syndrome: genetic insights into disease pathogenesis. Curr Opin Lipidol.

[14] Mohamed SM, Shalaby MA, El-Shiekh RA, El-Banna HA, Emam SR, Bakr AF (2023). Metabolic syndrome: risk factors, diagnosis, pathogenesis, and management with natural approaches. Food Chemistry Advances.

[15] Huang PL (2009). A comprehensive definition for metabolic syndrome. Dis Model Mech.

[16] Rus M, Crisan S, Andronie-Cioara FL, Indries M, Marian P, Pobirci OL, Ardelean AI (2023). Prevalence and risk factors of metabolic syndrome: a prospective study on cardiovascular health. Medicina (Kaunas).

[17] Yu E, Malik VS, Hu FB (2018). Cardiovascular disease prevention by diet modification: JACC Health Promotion Series. J Am Coll Cardiol.

[18] Bermudez V, Olivar LC, Torres W (2018). Cigarette smoking and metabolic syndrome components: a cross-sectional study from Maracaibo City, Venezuela. F1000Res.

[19] Magkos F, Yannakoulia M, Chan JL, Mantzoros CS (2009). Management of the metabolic syndrome and type 2 diabetes through lifestyle modification. Annu Rev Nutr.

[20] Jha BK, Sherpa ML, Imran M, Mohammed Y, Jha LA, Paudel KR, Jha SK (2023). Progress in understanding metabolic syndrome and knowledge of its complex pathophysiology. Diabetology.

[21] Das D, Shruthi NR, Banerjee A, Jothimani G, Duttaroy AK, Pathak S (2023). Endothelial dysfunction, platelet hyperactivity, hypertension, and the metabolic syndrome: molecular insights and combating strategies. Front Nutr.

[22] Moghadam-Ahmadi A, Soltani N, Ayoobi F (2023). Association between metabolic syndrome and stroke: a population based cohort study. BMC Endocr Disord.

[23] Senst B, Tadi P, Basit H, Jan A (2024). Hypercoagulability. StatPearls.

[24] Oktay AA, Paul TK, Koch CA (2000). Diabetes, cardiomyopathy, and heart failure. Endotext. MDText.com, Inc.: South Dartmouth.

[25] Guembe MJ, Fernandez-Lazaro CI, Sayon-Orea C, Toledo E, Moreno-Iribas C (2020). Risk for cardiovascular disease associated with metabolic syndrome and its components: a 13-year prospective study in the RIVANA cohort. Cardiovasc Diabetol.

[26] Chakraborty S, Verma A, Garg R, Singh J, Verma H (2023). Cardiometabolic risk factors associated with type 2 diabetes mellitus: a mechanistic insight. Clin Med Insights Endocrinol Diabetes.

[27] Manna P, Jain SK (2015). Obesity, oxidative stress, adipose tissue dysfunction, and the associated health risks: causes and therapeutic strategies. Metab Syndr Relat Disord.

[28] Guebre-Egziabher F, Alix PM, Koppe L, Pelletier CC, Kalbacher E, Fouque D, Soulage CO (2013). Ectopic lipid accumulation: A potential cause for metabolic disturbances and a contributor to the alteration of kidney function. Biochimie.

[29] Rezaianzadeh A, Namayandeh SM, Sadr SM (2012). National Cholesterol Education Program Adult Treatment Panel III Versus International Diabetic Federation Definition of Metabolic Syndrome, which one is associated with diabetes mellitus and coronary artery disease?. Int J Prev Med.

[30] Gundogan K, Bayram F, Gedik V (2013). Metabolic syndrome prevalence according to ATP III and IDF criteria and related factors in Turkish adults. Arch Med Sci.

[31] Roberts CK, Hevener AL, Barnard RJ (2013). Metabolic syndrome and insulin resistance: underlying causes and modification by exercise training. Compr Physiol.

[32] Liu PJ, Lou HP, Zhu YN (2020). Screening for metabolic syndrome using an integrated continuous index consisting of waist circumference and triglyceride: a preliminary cross-sectional study. Diabetes Metab Syndr Obes.

[33] Regufe VM, Pinto CM, Perez PM (2020). Metabolic syndrome in type 2 diabetic patients: a review of current evidence. Porto Biomed J.

[34] Grundy SM, Cleeman JI, Daniels SR (2005). Diagnosis and management of the metabolic syndrome: an American Heart Association/National Heart, Lung, and Blood Institute Scientific Statement. Circulation.

[35] Patel R, Sina RE, Keyes D (2024). Lifestyle modification for diabetes and heart disease prevention. StatPearls.

[36] Altawili AA, Altawili M, Alwadai AM (2023). An exploration of dietary strategies for hypertension management: a narrative review. Cureus.

[37] (2024). Physical activity guidelines for adults aged 19 to 64. nhs.uk. https://www.nhs.uk/live-well/exercise/physical-activity-guidelines-for-adults-aged-19-to-64/.

[38] Baker C, Retzik-Stahr C, Singh V, Plomondon R, Anderson V, Rasouli N (2021). Should metformin remain the first-line therapy for treatment of type 2 diabetes?. Ther Adv Endocrinol Metab.

[39] Aderinto N, Olatunji G, Kokori E, Olaniyi P, Isarinade T, Yusuf IA (2023). Recent advances in bariatric surgery: a narrative review of weight loss procedures. Ann Med Surg (Lond).

[40] Aguilar-Salinas CA, Viveros-Ruiz T (2019). Recent advances in managing/understanding the metabolic syndrome. F1000Res.

[41] Leon BM, Maddox TM (2015). Diabetes and cardiovascular disease: Epidemiology, biological mechanisms, treatment recommendations and future research. World J Diabetes.

[42] (2024). Healthy diet. https://www.who.int/news-room/fact-sheets/detail/healthy-diet.

[43] Mason JB, Sanders D, Musgrove P (2006). Community health and nutrition programs (chapter 56). Disease Control Priorities in Developing Countries, 2nd edition.

[44] Gorski MT, Roberto CA (2015). Public health policies to encourage healthy eating habits: recent perspectives. J Healthc Leadersh.

[45] Mamun A, Kitzman H, Dodgen L (2020). Reducing metabolic syndrome through a community-based lifestyle intervention in African American women. Nutr Metab Cardiovasc Dis.

[46] Kassi E, Pervanidou P, Kaltsas G, Chrousos G (2011). Metabolic syndrome: definitions and controversies. BMC Med.

[47] Saif-Ali R, Kamaruddin NA, Al-Habori M, Al-Dubai SA, Ngah WZ (2020). Relationship of metabolic syndrome defined by IDF or revised NCEP ATP III with glycemic control among Malaysians with type 2 diabetes. Diabetol Metab Syndr.

[48] Washington TB, Johnson VR, Kendrick K, Ibrahim AA, Tu L, Sun K, Stanford FC (2023). Disparities in access and quality of obesity care. Gastroenterol Clin North Am.

[49] Dalle Grave R, Calugi S, Centis E, Marzocchi R, El Ghoch M, Marchesini G (2010). Lifestyle modification in the management of the metabolic syndrome: achievements and challenges. Diabetes Metab Syndr Obes.

[50] Trang K, Grant SF (2023). Genetics and epigenetics in the obesity phenotyping scenario. Rev Endocr Metab Disord.

[51] Begum M, Choubey M, Tirumalasetty MB (2023). Adiponectin: a promising target for the treatment of diabetes and its complications. Life (Basel).

[52] Pomeroy A, Bates LC, Stoner L, Weaver MA, Moore JB, Nepocatych S, Higgins S (2022). Protocol for a longitudinal study of the determinants of metabolic syndrome risk in young adults. Transl J Am Coll Sports Med.

